# Supplementary material for: CSPG4-Specific CAR T Cells for High-Risk Childhood B Cell Precursor Leukemia
Source: Int J Mol Sci. 2019 Jun 5;20(11):2764. doi: 10.3390/ijms20112764 (PMC6600602; doi:10.3390/ijms20112764)
Supplement: Supplementary file 1 [file ijms-20-02764-s001.pdf]

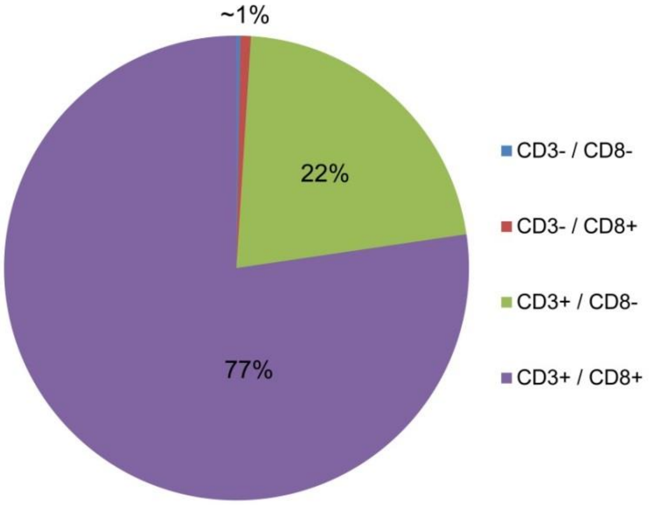

Supplemental figure S1: Cellular composition after T-cell expansion.
Healthy donor-derived bulk T cells were expanded using OKT3 and IL-2 as described in the Materials & Methods section. After 10 days, cellular composition was analyzed using double-staining for CD3 and CD8. Data are presented as means of 3 independent experiments.

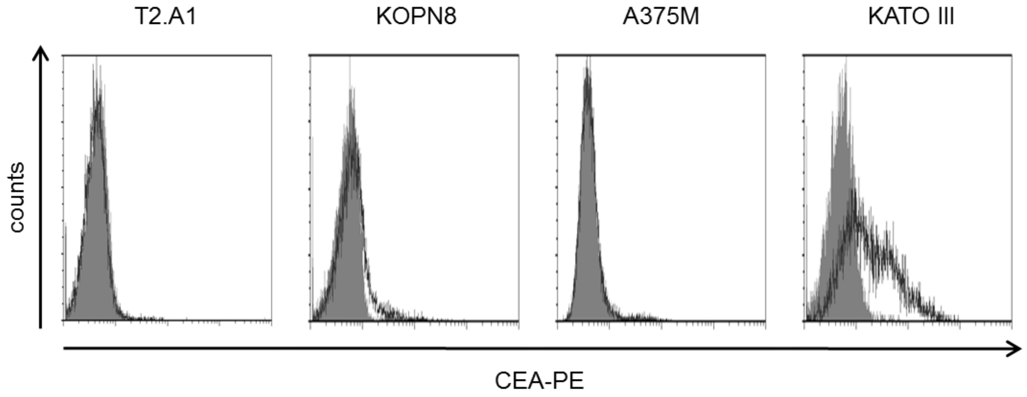

Supplemental figure S2: CEA expression on tumor cell lines.
Surface expression of CEA on T2.A1 cells, KOPN8 cells, and A375M cells in comparison to the CSPG4-positive Kato III cell line. One representative staining out of four independent experiments is presented.

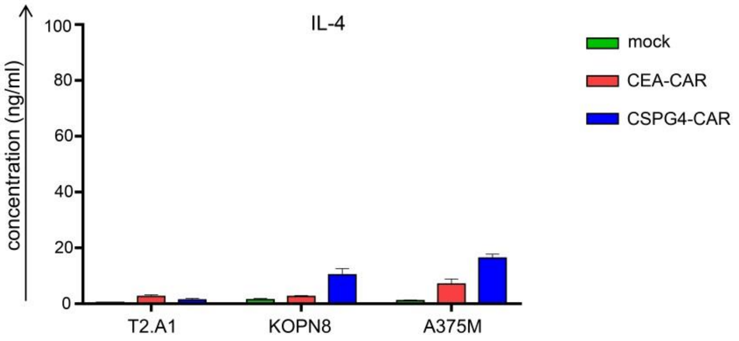

Supplemental figure S3: CSPG4-CAR T cells produce negligible quantities of IL-4.
The different T-cell conditions were generated as mentioned above (Fig. 2). Mock (no RNA) electroporated T cells and CEA-CAR T cells served as controls. Twenty-four hours after electroporation, T cells were co-incubated overnight at a 1.1 ratio with T2.A1 cells, KOPN8 cells, and A375M cells. Induced cytokine secretion was quantified in the supernatant with a cytometric bead array (CBA). Concentration of IL-4 is shown [pg/ml]. Data represent means ± SEM from 3 independent experiments.

Original data used for Figure S1

Percentage positive cells				Donor
CD3-/CD8-	CD3-/CD8+	CD3+/CD8-	CD3+/CD8+	
0.23	0.58	32.35	66.84	HD 1
0.17	0.45	21.03	78.35	HD 2
0.39	1.16	11.68	86.77	HD 3

Original data used for Figure 2c+d

	HD 1		
	CD69 (MFI)		
	**Mock**	**CEA-CAR**	**CSPG4-CAR**
**T2.A1**	2.11	8.83	11.82
**KOPN8**	0.28	4.14	21.84
**A375M**	0.05	10.37	22.41
	CD25 (MFI)		
	**Mock**	**CEA-CAR**	**CSPG4-CAR**
**T2.A1**	0	2.21	17.76
**KOPN8**	0	6.66	33.67
**A375M**	1.17	43.45	62.63
	HD 2		
	CD69 (MFI)		
	**Mock**	**CEA-CAR**	**CSPG4-CAR**
**T2.A1**	3.32	7.37	7.04
**KOPN8**	1.01	2.54	16.28
**A375M**	1.9	10.83	21.28
	CD25 (MFI)		
	**Mock**	**CEA-CAR**	**CSPG4-CAR**
**T2.A1**	2.71	5.36	4.76
**KOPN8**	1.22	4.91	17.51
**A375M**	4.96	19.42	50.88
	HD 3		
	CD69 (MFI)		
	**Mock**	**CEA-CAR**	**CSPG4-CAR**
**T2.A1**	0.99	8.87	12.37
**KOPN8**	0.1	2.51	19.87
**A375M**	0	10.29	21.25
	CD25 (MFI)		
	**Mock**	**CEA-CAR**	**CSPG4-CAR**
**T2.A1**	7.42	14.71	23.84
**KOPN8**	3.86	6.05	51.65
**A375M**	8.54	43.44	80.79

Original data used for Figure 3

HD 1				
**IL-2**	**TNF**	**IFNgamma**		
pg/ml	pg/ml	pg/ml		
81.6	1.0	3.0	T2.A1	**Mock**
25.8	6.5	465.8		**CEA-CAR**
91.7	6.1	99.5		**CSPG4-CAR**
33.4	21.5	166.9	KOPN8	**Mock**
13.6	29.6	1703.1		**CEA-CAR**
107.4	1690.5	9617.2		**CSPG4-CAR**
36.2	2.8	92.2	A375M	**Mock**
7.8	342.6	12909.2		**CEA-CAR**
3971.4	5252.5	18928.63		**CSPG4-CAR**
HD 2				
**IL-2**	**TNF**	**IFNgamma**		
pg/ml	pg/ml	pg/ml		
23.9	2.9	21.3	T2.A1	**Mock**
51.6	19.9	592.4		**CEA-CAR**
37.9	19.7	123.5		**CSPG4-CAR**
33.4	20.6	158.3	KOPN8	**Mock**
13.6	89.2	1330.7		**CEA-CAR**
107.4	1984.5	8631.0		**CSPG4-CAR**
36.2	5.1	63.2	A375M	**Mock**
55.7	263.9	7530.0		**CEA-CAR**
3115.9	3746.8	16607.7		**CSPG4-CAR**
HD 3				
**IL-2**	**TNF**	**IFNgamma**		
pg/ml	pg/ml	pg/ml		
28.5	2.3	0.3	T2.A1	**Mock**
14.2	5.2	200.9		**CEA-CAR**
24.1	6.8	83.0		**CSPG4-CAR**
55.9	20.8	177.4	KOPN8	**Mock**
14.1	45.8	581.6		**CEA-CAR**
103.8	1821.0	9038.8		**CSPG4-CAR**
10.9	11.1	59.8	A375M	**Mock**
32.6	150.4	7342.4		**CEA-CAR**
4785.6	6401.1	17481.73		**CSPG4-CAR**

Original data used for Figure S2

HD 1				
**IL-4**				
pg/ml				
0.6			T2.A1	**Mock**
3.2				**CEA-CAR**
2.2				**CSPG4-CAR**
2.1			KOPN8	**Mock**
3.0				**CEA-CAR**
9.3				**CSPG4-CAR**
1.7			A375M	**Mock**
10.5				**CEA-CAR**
16.4				**CSPG4-CAR**

HD 2				
**IL-4**				
pg/ml				
0.3			T2.A1	**Mock**
3.1				**CEA-CAR**
1.4				**CSPG4-CAR**
1.6			KOPN8	**Mock**
3.0				**CEA-CAR**
14.7				**CSPG4-CAR**
0.7			A375M	**Mock**
6.1				**CEA-CAR**
18.8				**CSPG4-CAR**

HD 3				
**IL-4**				
pg/ml				
0.5			T2.A1	**Mock**
1.6				**CEA-CAR**
0.5				**CSPG4-CAR**
0.7			KOPN8	**Mock**
1.8				**CEA-CAR**
7.1				**CSPG4-CAR**
1.0			A375M	**Mock**
4.5				**CEA-CAR**
13.8				**CSPG4-CAR**

Original data used for Figure 4a

	HD 1		
	degranulation (%)		
	**Mock**	**CEA-CAR**	**CSPG4-CAR**
T2.A1	1.4	4.0	15.0
KOPN8	1.8	3.7	21.1
A375M	1.4	12.7	42.4
	HD 2		
	degranulation (%)		
	**Mock**	**CEA-CAR**	**CSPG4-CAR**
T2.A1	2.0	4.6	7.3
KOPN8	2.2	3.9	21.7
A375M	1.1	6.7	39.7
	HD 3		
	degranulation (%)		
	**Mock**	**CEA-CAR**	**CSPG4-CAR**
T2.A1	1.2	3.5	18.9
KOPN8	2.7	4.4	27.0
A375M	1	12.3	42.2

Original data used for Figure 4b+c

HD 1				
Lysis(%)				
60:1	20:1	6:1	2:1	
5.0	5.3	5.0	4.3	T2.A1 Mock
11.5	12.6	8.3	7.8	T2.A1 CEA-CAR
13.8	13.6	12.5	6.0	T2.A1 CSPG4-CAR
5.6	8.1	7.0	9.9	KOPN8 mock
36.0	24.0	17.2	7.4	KOPN8 CEA-CAR
53.3	53.3	44.8	39.2	KOPN8 CSPG4-CAR

HD 2				
Lysis(%)				
60:1	20:1	6:1	2:1	
24.3	10.3	2.9	0	T2.A1 Mock
18.2	7.2	3.3	0	T2.A1 CEA-CAR
21.0	8.1	3.2	3.3	T2.A1 CSPG4-CAR
18.8	10.0	11.9	6.4	KOPN8 mock
40.8	23.9	11.3	9.6	KOPN8 CEA-CAR
45.7	55.1	36.0	40.1	KOPN8 CSPG4-CAR

HD 3				
Lysis(%)				
60:1	20:1	6:1	2:1	

26.6	12.6	4.7	1.6	T2.A1 Mock
18.5	10.1	4.6	2.5	T2.A1 CEA-CAR
24.8	18.7	8.3	7.3	T2.A1 CSPG4-CAR
24.2	20.5	18.6	18.2	KOPN8 mock
56.6	46.0	37.0	30.3	KOPN8 CEA-CAR
81.7	95.3	97.3	64.2	KOPN8 CSPG4-CAR
